# Carboxyhemoglobin predicts oxygenator performance and imminent oxygenator change in extracorporeal membrane oxygenation

**DOI:** 10.1186/s40635-024-00626-7

**Published:** 2024-04-24

**Authors:** Rolf Erlebach, Alix Buhlmann, Rea Andermatt, Benjamin Seeliger, Klaus Stahl, Christian Bode, Reto Schuepbach, Pedro David Wendel-Garcia, Sascha David, the BonHanZA (Bonn-Hannover-Zurich-ARDS) study group, Eva-Maria Kleinert, Daniel Andrea Hofmaenner, Mattia M Müller, Christoph Camille Ganter, Tobias Welte, Thorben Pape, Ann-Kathrin Rath, Bahar Nalbant, Jannik Ruwisch, Christian Putensen, Konrad Peukert, Andrea Sauer, Lennart Wild

**Affiliations:** 1 Institute of Intensive Care Medicine, University Hospital Zurich, Zurich, Switzerland; 2 Department of Respiratory Medicine, Hannover Medical School, Hannover, Germany; 3 Department of Gastroenterology, Hepatology, Infectious Diseases and Endocrinology, Hannover Medical School, Hannover, Germany; 4 Department of Anesthesiology and Intensive Care Medicine, University Hospital Bonn, Bonn, Germany

**Keywords:** Hemolysis, Thrombosis, Blood gas analysis, Co-oximetry, Coagulation, Surveillance

## Abstract

**Background:**

The continuous exposure of blood to a non-biological surface during extracorporeal membrane oxygenation (ECMO) may lead to progressive thrombus formation in the oxygenator, hemolysis and consequently impaired gas exchange. In most centers oxygenator performance is monitored only on a once daily basis. Carboxyhemoglobin (COHb) is generated upon red cell lysis and is routinely measured with any co-oximetry performed to surveille gas exchange and acid–base homeostasis every couple of hours. This retrospective cohort study aims to evaluate COHb in the arterial blood gas as a novel marker of oxygenator dysfunction and its predictive value for imminent oxygenator change.

**Results:**

Out of the 484 screened patients on ECMO 89, cumulatively requiring 116 oxygenator changes within 1833 patient days, including 19,692 arterial COHb measurements were analyzed. Higher COHb levels were associated with lower post-oxygenator pO_2_ (estimate for log(COHb): − 2.176 [95% CI − 2.927, − 1.427], *p* < 0.0001) and with a shorter time to oxygenator change (estimate for log(COHb): − 67.895 [95% CI − 74.209, − 61.542] hours, *p* < 0.0001). COHb was predictive of oxygenator change within 6 h (estimate for log(COHb): 5.027 [95% CI 1.670, 15.126], *p* = 0.004).

**Conclusion:**

COHb correlates with oxygenator performance and can be predictive of imminent oxygenator change. Therefore, longitudinal measurements of COHb in clinical routine might be a cheap and more granular candidate for ECMO surveillance that should be further analyzed in a controlled prospective trial design.

**Supplementary Information:**

The online version contains supplementary material available at 10.1186/s40635-024-00626-7.

## Background

Extracorporeal membrane oxygenation (ECMO) provides temporary organ support to patients with respiratory or circulatory failure. Despite increased use of ECMO in the past decade, there remains a wide knowledge gap for major aspects of clinical practice [1]. The ECMO circuit includes a blood pump, an artificial gas exchanger (called oxygenator) and conduit tubing [2]. Change of the ECMO circuit mostly due to performance issue of the oxygenator is required in up to 31% of patients [3–5], mainly caused by ECMO-associated coagulopathies, such as oxygenator thrombosis, hemolysis, hyperfibrinolysis and consecutive bleeding [4–6]. During circuit change, patients are temporarily cut off extracorporeal support, which includes the risk for hypoxemia, hemodynamic instability, blood loss, and even air embolism. Routine monitoring of consumptive coagulopathy, hemolysis, circuit pressures and gas transfer is recommended to anticipate oxygenator failure [5, 7, 8], prevent emergency situations such as acute pumphead thrombosis [6] and allow elective circuit change.

Mechanisms of hemolysis during ECMO support include thrombus formation and excessive negative suction pressure leading to cavitation and shear stress [7, 9–11]. Progressive thrombus generation in the oxygenator with consequent hemolysis, obstruction to flow and impaired gas change may be limited and reversed by replacing oxygenator [6, 7]. To identify a dysfunctional oxygenator as early as possible, most centers rely on (heterogeneous) local surveillance protocols including visual circuit inspection, extensive daily blood tests (e.g., d-dimers, fibrinogen, LDH, bilirubin, plasma free Hb, etc.) along with oxygenator performance tests measuring post-oxygenator pO_2_ (PpostO_2_) under maximal oxygen support. Nevertheless, granularity of these tests is low as these values are most commonly measured only once daily and more dynamic parameters might therefore be highly desirable.

During red blood cell lysis, plasma free hemoglobin (fHb) is degraded to free heme in a nitric oxide (NO)-consumptive reaction. Heme oxygenase further metabolizes heme into biliverdin, free iron, and carbon monoxide [12, 13]. Carbon monoxide (CO) then binds with high affinity to hemoglobin (about 250-fold greater as with oxygen) [14, 15]. COHb is a promising parameter of overall oxygenator function because it is routinely measured every few hours, and both its production and its elimination are theoretically dependent on oxygenator condition [15]. We therefore analyzed the potential use of COHb as a novel parameter predicting oxygenator dysfunction

## Methods

### Design and study population

This retrospective cohort study aimed to evaluate COHb as a marker of oxygenator dysfunction and performance. All patients that received respiratory and/or circulatory support via ECMO with at least one oxygenator change since the electronical health care record system was established in our tertiary intensive care unit were included (starting 2018 until 2021). Patients with a documented refusal of further use of anonymized health-related data were excluded. The cantonal ethics commission of Zurich approved the study protocol (ZH 2022-00163) and the procedures were followed according to the ethical standards of the regional committee on human experimentation and with the Helsinki Declaration of 1975.

### Institutional policies

In our institution, indication for ECMO implantation is discussed with the designated ECMO team consisting of an intensivist, a cardiac surgeon, a perfusionist and a cardiac anesthetist experienced in transesophageal echocardiography. The ECMO team performs implantation around the clock.

Routine monitoring of ECMO function is performed by the responsible nurse and overseen by the treating intensivist. This includes visual inspection of correct ECMO installation and thrombus formation in the circuit every 8 h. Peripheral oxygen saturation (SpO2) is monitored with continuous pulse oximetry and arterial blood gas analysis every 2–4 h. Oxygenator performance is evaluated once daily by measuring post-oxygenator partial pressure of oxygen (pO2) on maximum support [fraction of delivered oxygen by the sweep gas (F_s_O_2_) = 100%]. Markers of consumptive coagulopathy and hyperfibrinolysis [D-dimer, fibrinogen, platelets, international normalized ratio (INR), partial thromboplastin time (PTT), thrombin time (TT)] and hemolysis [hemoglobin, bilirubin, lactate dehydrogenase (LDH)] are measured at least daily. Thromboelastography is only used in specific cases mostly when the clinical and chemical situation is suggestive for hyperfibrinolysis.

Unfractionated heparin is used for anticoagulation unless contraindicated. Our in-house targets of anticoagulation are an anti-FXa activity of 0.2–0.3 IU/ml for V-V ECMO (0.3–0.4 before 2021) and 0.3–0.4 IU/ml for V-A ECMO (initially measured every 6 h, then in stable conditions twice daily) unless the patient has an indication for higher target values.

Indication for oxygenator replacement at our institution are prolongation of ECMO support and at least one of the following criteria:

- Progressive circuit-related coagulopathy including hyperfibrinolysis as evidenced by rising d-dimers, decreased fibrinogen, platelets, and abnormal standard coagulation tests (INR, PTT, TT) AND/OR.
- Progressive hemolysis assessed by hemoglobin, LDH and bilirubin AND/OR.
- Worsening gas exchange evaluated by reduced oxygen uptake by the membrane oxygenator (post-oxygenator pO_2_ < 20 kPa at F_s_O_2_ = 100%).

Are any of these indicators positive, we discuss a potential oxygenator change interdisciplinary between a senior intensivist, perfusionist and cardiac surgeon, considering gas transfer capability, hematologic profile (including evaluation of other causes) and pressure indices. If an oxygenator change is performed, the entire circuit is replaced except for a short segment of tubing on the side of the patient to connect the new circuit.

Those institutional policies did not change throughout the studied period.

### Variables and data acquisition

Data were extracted from the local clinical information system and the patient data and monitoring system for the duration of ECMO therapy. Population characteristics before ECMO implantation were collected. In case of transition from cardiopulmonary bypass (CPB) to ECMO, values before CPB were chosen. Mortality was assessed until ICU discharge. Co-oximetry was performed with ABL825 FLEXQ (Radiometer, Denmark). For the purpose of data modeling, co-oximetrically measured parameters were summarized to a 6-hourly average, whereas daily-measured laboratory parameters were assumed representative for 24 h in case no updated measurement was available.

### Statistical analysis

Variable distributions including temporal distributions were assessed by visual inspection of histograms and quantile–quantile plots. Non-linearly distributed variables were modeled by either logarithmic transformation or natural cubic splines, as best fitting. In order to ensure a well-defined causal model to study the potential causal relationship between COHb and oxygenator performance, a structural causal model was graphically specified employing directed acyclic graphs based on a pathophysiological consensus of all investigators (Additional file 1: Figure S1).

The time-varying association between COHb and oxygenator function was modeled by means of univariable and multivariable hierarchical linear mixed-effects models. The post-oxygenator partial pressure of oxygen was considered as dependent variable and COHb, as well as time, including their interaction, as independent variables. Furthermore, hierarchically clustered per-oxygenator-in-patient random intercepts and intra-oxygenator-in-patient random slopes were entered into the model. Finally, all variables specified in the directed acyclic graph were implemented as time-varying covariates for the multivariable version of the model. Analogously, the time to oxygenator change was modeled by means of hierarchical linear mixed-effects models, and the probability of oxygenator change through hierarchical generalized linear mixed-effects models of the binomial family.

The time-to-event analysis considering oxygenator change as outcome of interest was performed by means of univariable and multivariable mixed-effects time-varying Cox proportional hazards models including a hierarchical frailty term of oxygenator clustered within patient.

Statistical analysis was performed through a fully scripted data management pathway using the R environment for statistical computing version 4.3.1. A two-sided *p* < 0.05 was considered statistically significant. Values are given as means with standard deviations, medians with interquartile ranges or counts with percentages, as appropriate.

## Results

### Population characteristics

In total, 484 ECMO patients were screened, of whom 89 required one or more oxygenator changes and were consequently included into the final cohort. Thirty-three (37%) patients initially received ECMO in V-V configuration and 56 (63%) in V-A configuration. Patients were mainly male (71%) of median age 54 [IQR 44-60] years. Median SOFA score at the time of cannulation was 10 [IQR 8–12] and overall ICU mortality was 58%. Cumulatively, 116 oxygenator changes were detected over 1833 patient days and 19,692 arterial COHb measurements (median per patient 206 [IQR 144–275]) were performed. Population characteristics are summarized in Table 1. The median duration on ECMO support was 17.9 days [IQR 12.5, 24.7] and the median time to oxygenator exchange was 9.3 days [IQR 6.3, 18.0]. Median FCOHb was 2.0 [IQR 1.5–2.7] % overall, 2.4 [IQR 1.7–3.3] % in V-V ECMO and 1.7 [IQR 1.3–2.2] % in V-A ECMO (*p* < 0.0001). A histogram visualizing the distribution of COHb is presented in Additional file 1: Figure S2.

**Table 1:** Population characteristics at time of ECMO implantation

Variable	Overall
*n*	89
Age, years	54 [44, 60]
Sex	
Male	63 (71)
Female	26 (29)
BMI, kg/m^2^	26.8 [23.5, 30.8]
ECMO configuration	
V-V	33 (37)
V-A	56 (63)
Main diagnosis	
Sepsis	44 (49)
Cardiogenic shock	51 (57)
ARDS	38 (43)
Comorbidities	
Obesity	25 (28)
Cardiovascular disease	42 (47)
COPD	5 (6)
Chronic kidney disease	10 (11)
Diabetes mellitus	20 (22)
Cancer	9 (10)
Mechanical ventilation	76 (85)
Renal replacement therapy	9 (10)
Norepinephrine, µg/min	12 [4, 35]
PEEP, mbar	7 [5, 12]
PaO_2_/FiO_2_ at cannulation, mmHg	89 [62, 149]
Bilirubin, µmol/L	10 [6, 16]
LDH, U/L	672 [435, 1130]
Platelets, G/L	210 [123, 282]
Fibrinogen, g/L	4.3 [2.2, 6.4]
d-Dimer, mg/L	3.5 [2.2, 5.1]
pH	7.27 [7.14, 7.38]
PaCO_2_, kPa	6.1 [4.8, 9.0]
PaO_2_, kPa	10.3 [8.4, 13.8]
Hemoglobin, g/L	104 [87, 124]
SaO_2_, %	93 [89, 97]
*F*O_2_Hb, %	90 [88, 95]
*F*COHb, %	0.9 [0.6, 1.3]
*F*MetHb, %	0.9 [0.7, 1.3]
Lactate, mmol/L	1.9 [1.3, 5.0]

Numbers are presented as median [interquartile range] or count (%)

*ARDS* acute respiratory distress syndrome, *BMI* body mass index, *COPD* chronic obstructive pulmonary disease, *ECMO* extracorporeal membrane oxygenation, *FiO*_*2*_ fraction of inspired oxygen, *FCOHb* fraction of total hemoglobin (ctHb) that is present as carboxyhemoglobin (COHb), *FMetHb* fraction of total hemoglobin (ctHb) that is present as methemoglobin (MetHb),* FO*_*2*_*Hb* fraction of total hemoglobin (ctHb) that is present as oxyhemoglobin (O_2_Hb), *LDH* lactate dehydrogenase, *PaCO*_*2*_ partial pressure of carbon dioxide in arterial blood, *PEEP* positive end-expiratory pressure, *PaO*_*2*_ partial pressure of oxygen in arterial blood, *SaO*_*2*_ oxygen saturation in arterial blood, *SpO*_*2*_ peripheral oxygen saturation (pulse oximetry), *V-V* veno-venous, *V-A* veno-arterial

### Oxygenator performance

**Fig. 1:**
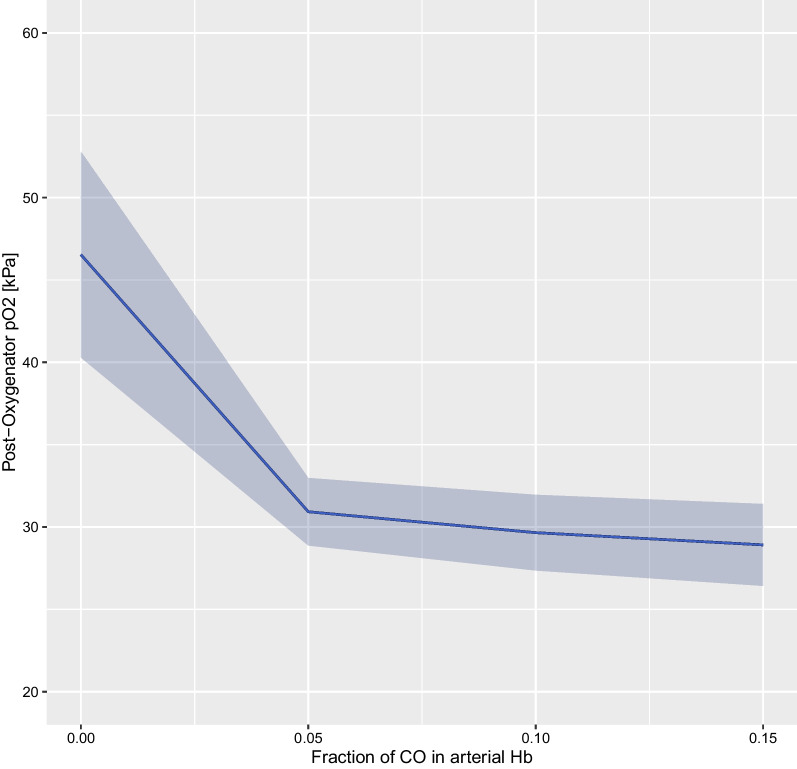
Association of COHb with oxygenator performance (post-oxygenator pO_2_) based on an unadjusted hierarchical linear mixed-effects model of log(COHb) on PpostO_2_—COHb expressed as fraction of total Hb. COHb was logarithmically transformed for modeling purposes, and backtransformed for plotting purposes in order to improve interpretability

Higher COHb levels were associated with worse oxygenator performance represented by PpostO_2_ in a hierarchical linear mixed-effects model (estimate for log(COHb): − 2.176 [95% CI − 2.927, − 1.427], *p* < 0.0001), visualized in Fig. 1. The negative effect remained after adjustment for co-factors (*p* = 0.0366) (Table 2).

**Table 2:** Adjusted model of oxygenator performance based on COHb

Fixed-effects	Estimate	Std. error	*p*-value
Intercept	37.600	1.950	< 0.0001
log(COHb)	− 0.658	0.315	0.0366
Time, h	− 0.031	0.004	< 0.0001
LDH, U/L	0.000	0.000	0.8554
Bilirubin, µmol/L	0.009	0.004	0.0140
Hemoglobin, g/L	0.000	0.000	0.0669
Sweep gas flow, L/min	0.462	0.070	< 0.0001
Fibrinogen, g/L	0.052	0.133	0.6968
D-dimer, mg/L	− 0.722	0.058	< 0.0001
Blood flow, L/min	− 0.016	0.015	0.2804
Lactate, mmol/L	0.541	0.102	< 0.0001
Norepinephrine, µg/min	− 0.032	0.030	0.2971

Adjusted hierarchical linear mixed-effects model of log(COHb) on PpostO_2_

*LDH* lactate dehydrogenase

### Oxygenator change

**Fig. 2:**
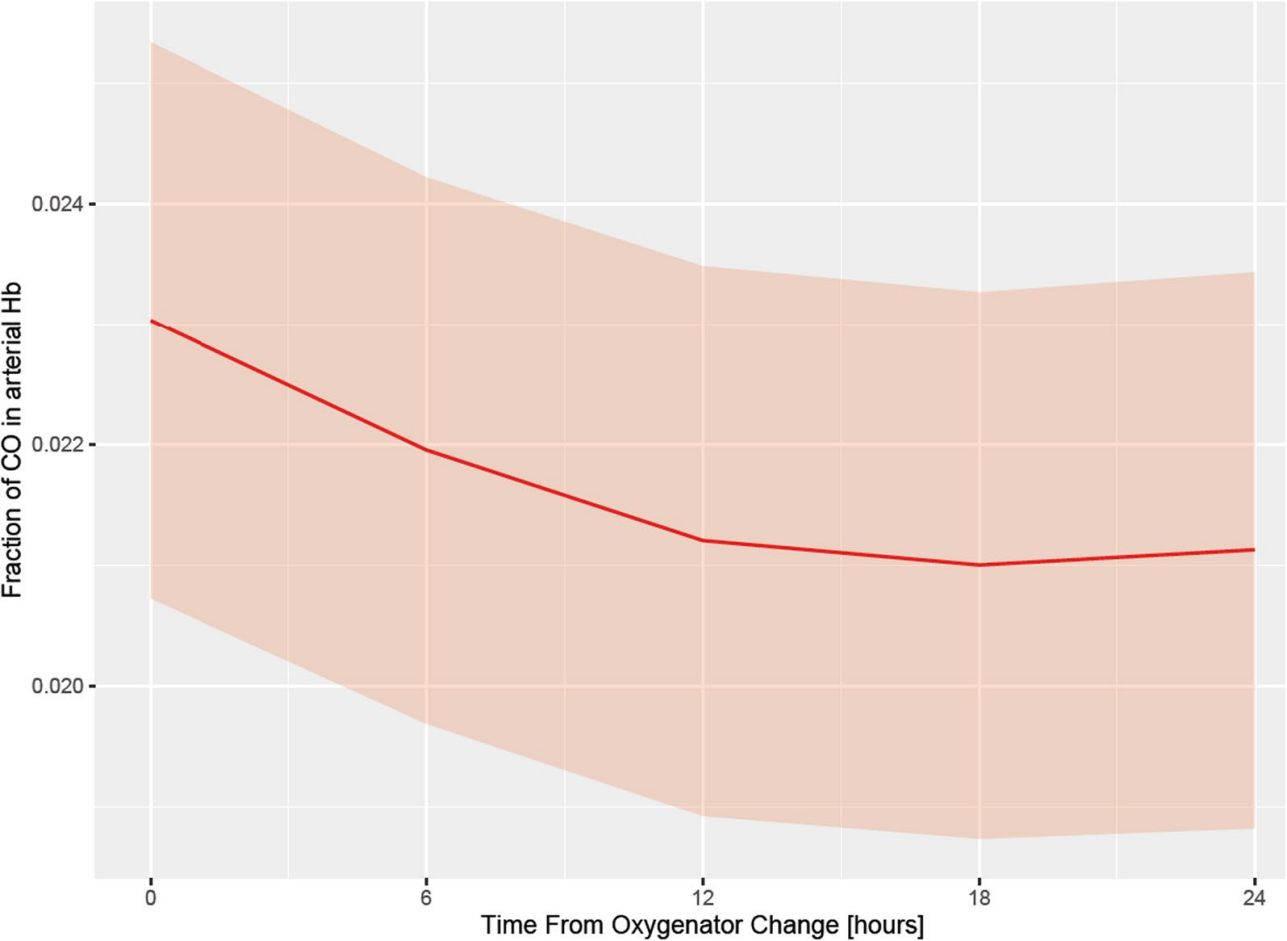
Association of time after oxygenator change and COHb based on an unadjusted hierarchical linear mixed-effects model of log(COHb)—COHb expressed as fraction of total Hb. COHb was logarithmically transformed for modeling purposes, and backtransformed for plotting purposes in order to improve interpretability

Levels of COHb within 6 h after oxygenator change were lower than within 6 h before replacement (estimate: − 0.0023 [95% CI − 0.0041, − 0.0005], *p* = 0.0106), indicating that a change of a dysfunctional oxygenator led to a reduction of before elevated COHb. We found that COHb level declined until about 12 h, until—in some cases—another oxygenator dysfunction occurred, which again led to rising levels (Fig. 2).

**Fig. 3:**
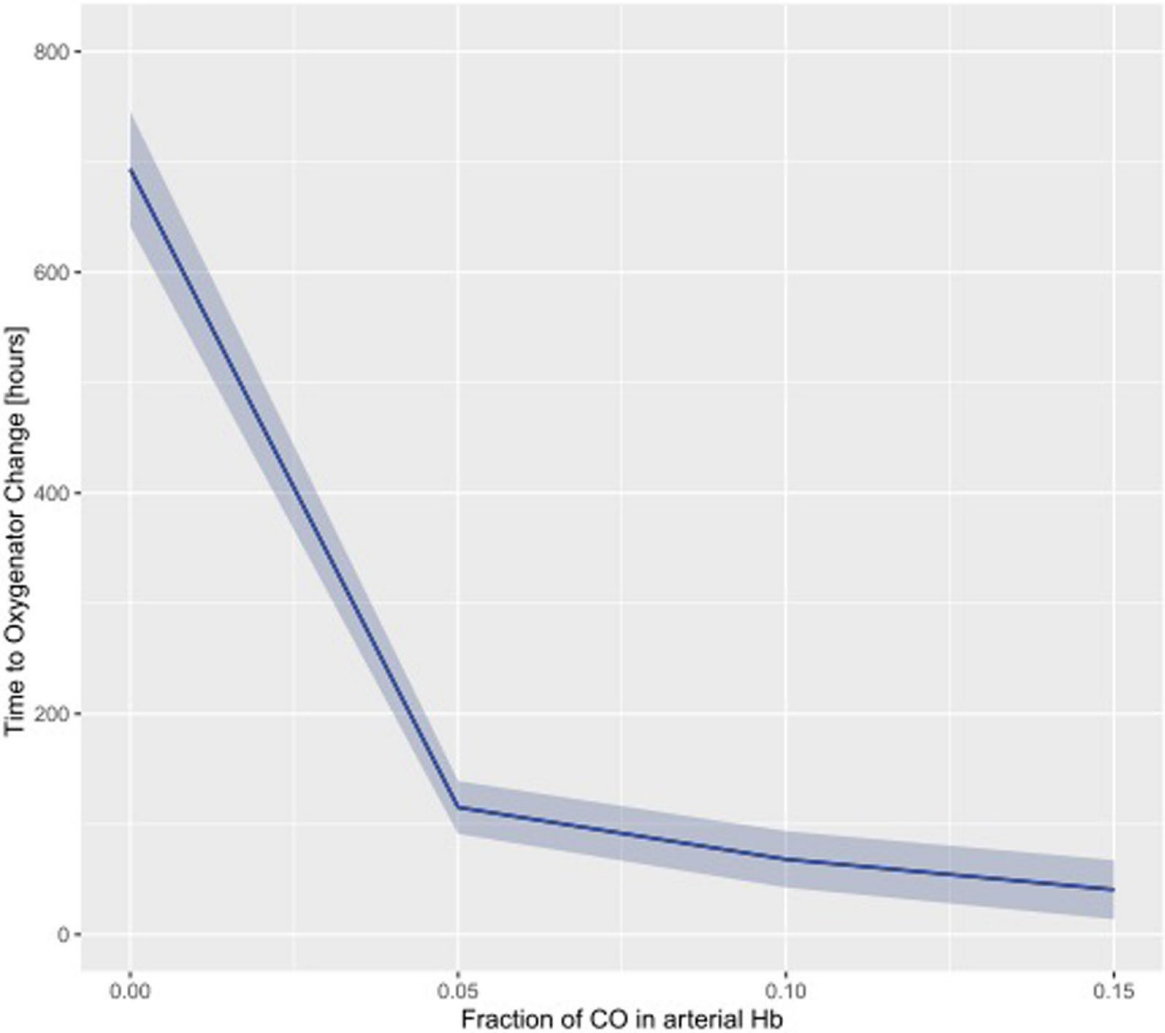
Association of COHb with time to oxygenator change based on an unadjusted hierarchical linear mixed-effects model of log(COHb)—COHb expressed as fraction of total Hb. COHb was logarithmically transformed for modeling purposes, and backtransformed for plotting purposes in order to improve interpretability

Higher COHb levels were associated with a shorter time to oxygenator change in the unadjusted (Estimate: − 67.895 [95% CI − 74.209, − 61.542], *p* < 0.0001) (Fig. 3) and in the adjusted model (*p* < 0.0001) (Table 3).

**Table 3:** Adjusted model of time to oxygenator change based on COHb

Fixed-effects	Estimate	Std. error	*p*-value
Intercept	113.000	66.500	0.090
log(COHb)	− 37.700	8.850	< 0.0001
Time, h	− 0.001	0.002	0.555
LDH, U/L	− 1.370	0.099	< 0.0001
Bilirubin, µmol/L	0.000	0.000	0.497
Hemoglobin, g/L	4.100	1.820	0.024
Sweep gas flow, L/min	20.500	3.600	< 0.0001
Fibrinogen, g/L	− 18.200	1.530	< 0.0001
D-dimer, mg/L	0.666	0.461	0.148
Blood flow, L/min	12.400	2.920	< 0.0001
Lactate, mmol/L	0.343	1.250	0.784

Adjusted hierarchical linear mixed-effects model of log(COHb) on time to oxygenator change (h)

*LDH* lactate dehydrogenase

**Fig. 4:**
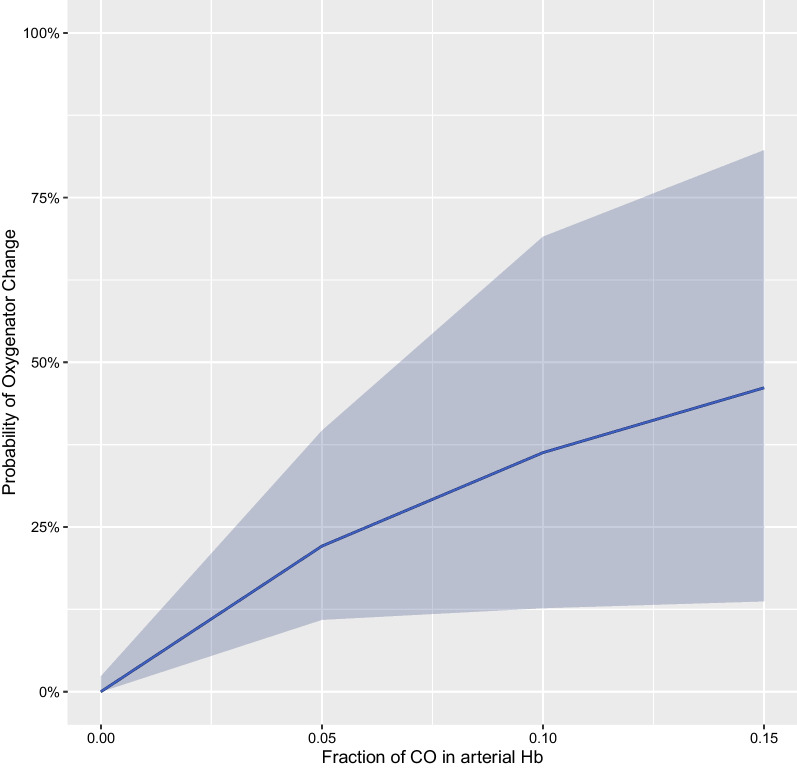
Probability of oxygenator change within 6 h based on COHb (expressed as fraction of total Hb), modeled by means of a hierarchical generalized linear mixed-effects model. Odds ratio for log(COHb): 5.027 [95% CI 1.670, 15.126], *p* = 0.004. COHb was logarithmically transformed for modeling purposes, and backtransformed for plotting purposes in order to improve interpretability

**Fig. 5:**
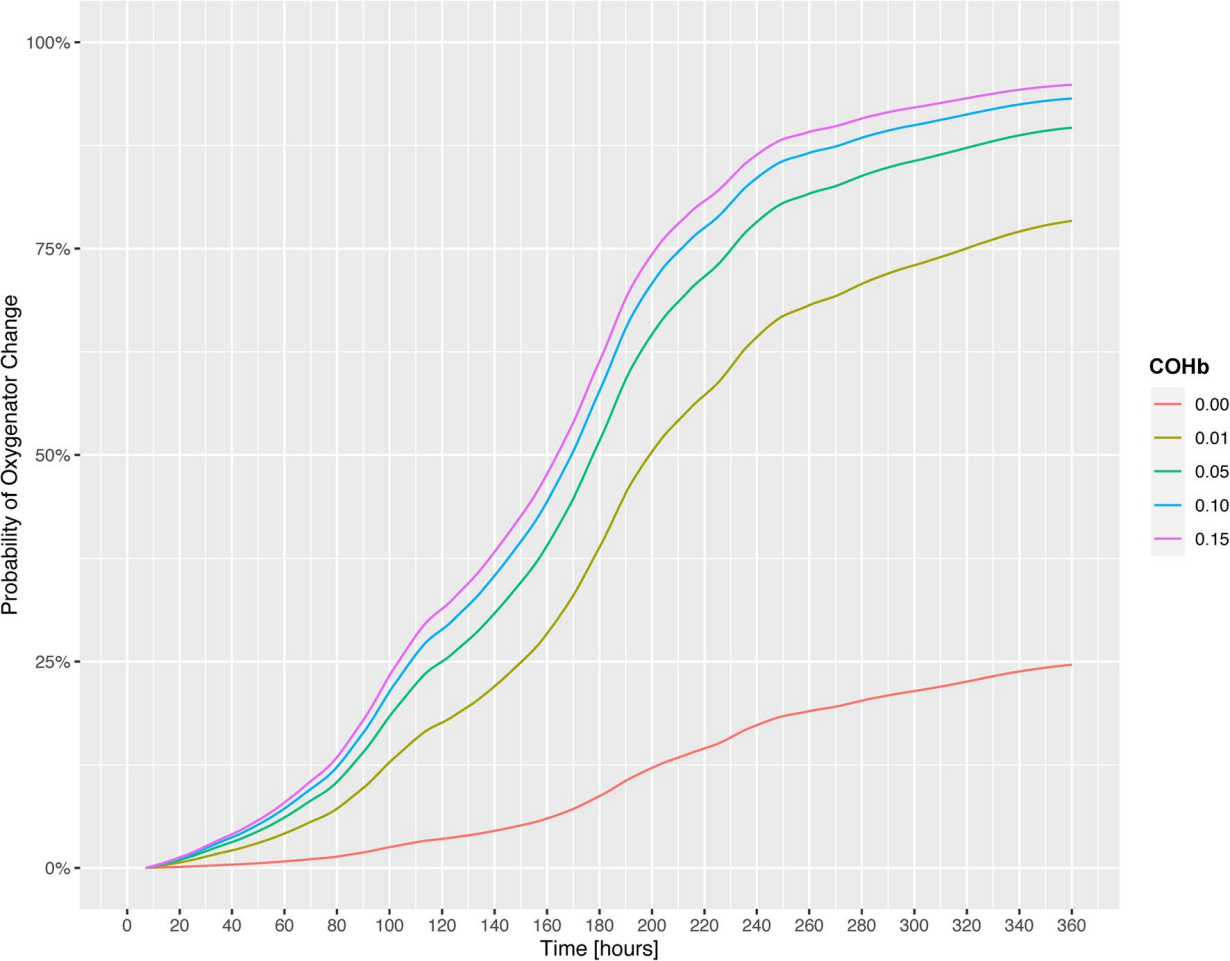
Cumulative probability of oxygenator change (within 6 h) based on time from COHb measurement and grouped by COHb levels (hierarchical multilevel mixed-effects time-varying Cox proportional hazards model)

The predictive ability of COHb on oxygenator change within 6 h was evaluated with a hierarchical generalized linear mixed-effects model of the binomial family and a hierarchical mixed-effects time-varying Cox proportional hazards model. In the unadjusted generalized linear model, the odds ratio for oxygenator change within 6 h based on log(COHb) was 5.027 [95% CI 1.670, 15.126],* p* = 0.004 (Fig. 4). Similarly, in the Cox proportional hazards model, COHb was associated with a higher probability of oxygenator change within 6 h (hazard ratio for log(COHb): 1.277 [95% CI 1.188, 1.373],* p* < 0.0001 (Fig. 5). Results of the adjusted models are presented in Table 4.

**Table 4:** Adjusted models for prediction of oxygenator change based on COHb

Fixed-effects	Logistic regression model			Cox regression model		
	Estimate	Std. error	*p*-value	Estimate	Std. error	*p*-value
Intercept	4.50E−01	1.36E+00	< 0.0001			
log(COHb)	8.08E−01	3.26E−01	0.013301	1.97E−01	2.46E−01	< 0.0001
Time, h	− 1.21E−02	4.15E−03	< 0.0001			
LDH, U/L	3.40E−05	3.36E−05	0.31158	5.89E−05	5.10E−05	0.00236
Bilirubin, µmol/L	1.37E−03	9.39E−04	1.45E−01	1.72E−03	1.80E−03	0.00341
Hemoglobin, g/L	5.69E−07	9.21E−06	9.51E−01	− 3.50E−02	1.66E−02	< 0.0001
Sweep gas flow, L/min	1.07E−02	3.55E−02	7.63E−01	− 7.04E−03	4.31E−02	0.61313
Fibrinogen, g/L	− 7.75E−02	5.50E−02	1.59E−01	− 1.62E−01	7.93E−02	< 0.0001
D-dimer, mg/L	7.81E−02	2.30E−02	6.79E−04	6.27E−02	3.71E−02	0.03171
Blood flow, L/min	− 2.41E−01	9.17E−02	8.67E−03	− 6.27E−04	1.45E−02	0.89013
Lactate, mmol/L	1.16E−01	4.85E−02	1.64E−02	1.48E−03	4.16E−03	0.43365
Intercept	6.38E−03	3.34E−03	5.62E−02	1.13E−01	8.11E−02	< 0.0001

Adjusted hierarchical generalized linear mixed-effects model of the binomial family (left) and hierarchical mixed-effects time-varying Cox proportional hazards model (right) of log(COHb) on oxygenator change within 6 h

*LDH* lactate dehydrogenase

## Discussion

In this retrospective cohort study of patients on ECMO requiring oxygenator change(s), high COHb levels were associated with worse oxygenator performance, predictive of oxygenator change within the next 6 h and associated with a shorter time to oxygenator change.

In general, COHb levels seem to be slightly elevated in critically ill patients [16–18], but can substantially increase during ECMO support [19–26]. Their prognostic value has not been fully understood. While some studies suggest a better ICU survival with higher COHb levels in medical ICU patients [27], others state a worse survival in critically ill children [28]. In ECMO patients, a COHb level > 2% after 24 h on extracorporeal life support was associated with a higher in-hospital mortality and peak COHb was shown to be an independent predictor of death [29]. Our results confirm overall high COHb levels in patients on ECMO (median arterial COHb 2.0 [IQR 1.5–2.7] %).

Several factors other than device-related hemolysis can lead to accumulation of COHb in ECMO patients. First, heme-oxygenase-1 (HO-1) is inducible by other factors than heme. Oxidative stress readily induces HO-1, especially in the lungs [12, 13], and may partially explain why elevated COHb levels were more frequent in V-V ECMO than V-A ECMO in our study and in previous reports [30, 31]. Likewise, nitric oxide is a potent inducer of HO-1 [12, 13] and treatment with inhalative NO (iNO) has been associated with increased COHb levels [17]. At our institution, iNO therapy is usually performed in severe ARDS patients as an attempt to avoid ECMO and is consequently weaned in the early phase of a running ECMO. We also use iNO in severe right heart failure and pulmonary hypertension, but these cases are a true minority of the current cohort. Therefore, this should not represent a relevant cofactor in this study. Second, hemolysis outside the ECMO circuit could have influenced the results, which we cannot exclude. However, hemolysis inside the circuit is a well-known complication of ECMO with a prevalence ranging from 2 to 67% and the most likely cause in this setting. Third, up to 14% of endogenous CO arise from other sources than heme degeneration [12], which are only partially understood and not explored in this analysis. We consider hemolysis inside the ECMO circuit together with reduced pulmonary elimination due to marginal lung function (V-V ECMO) or bypassing of the lung (V-A ECMO), as the most likely cause of rising COHb levels in our patients. The effectiveness of ECMO in reducing moderately elevated COHb is unclear but the residence time inside the oxygenator may be too short [15, 32]. This is probably more important with high blood flow rates in patients already hypoxemic [31]. In this setting, an only minimally reduced oxygenator performance may have a large impact on COHb elimination. Therefore, the combination of rising COHb levels and a reduced oxygenator performance point to a failing oxygenator.

Timely recognition of relevant hemolysis in ECMO patient can be crucial. Classical markers of hemolysis such as LDH, bilirubin and haptoglobin all suffer from lack of specificity and are measured away from the patient in a laboratory. fHb as the proposed gold standard has also some limitations, as it is not readily available everywhere, spectrophotometry takes time and furthermore the results are often influenced by high bilirubin and lipid levels or traumatic sampling [33].

By contrast, COHb is part of routinely collected blood gases and thus widely and rapidly available. It has been demonstrated that COHb as a simple point of care (POC) measurement correlates with the more complex fHb and other markers of hemolysis [20, 24, 29]. Moreover, studies proposed a COHb level > 2% as a threshold for the detection of hemolysis (sensitivity 85%, specificity 86%) and a fraction of > 2.7% had a specificity of even 99% in a critically ill population with 50% of patients having hemolytic anemia [34]. Several studies did not only show a progressive COHb increase over time with a higher COHb peak for longer ECMO runtime and higher blood velocity [23, 25, 29, 31], but also a significant decrease after oxygenator change [21, 24, 35]. Moreover, no correlation between COHb and oxygenator lifespan in general was shown, which additionally suggests that a well working oxygenator does not raise COHb [35]. In this retrospective study, COHb values were obviously not blinded to the physicians who evaluated the change of an oxygenator but the potential usefulness of COHb in this context was at that time not known to our team thereby providing sort of a natural blinding process due to ignorance.

Our study has limitations. First, we only included patients with an oxygenator change and hence cannot compare COHb values with other patients on ECMO that did not require any oxygenator change. Nevertheless, we modeled our data by means of time-varying mixed-effects models, enabling patients to function as their own temporal. Second, although indications of oxygenator change are more or less standardized by our in-house SOP, this study is still a retrospective analysis thereby subjective to bias. Furthermore, patients that have already recovered from their initial organ failure may be rapidly weaned from a dysfunctional ECMO rather than exchanging the oxygenator a few hours before the ECMO would be explanted anyway. We did not routinely collect indications for oxygenator change, but other studies [5, 6] showed that worsening gas transfer capability and device-related coagulation disorders were the most frequent attributed causes for oxygenator changes. Monitoring of gas change, hemolysis, coagulopathy and pressure changes allowed identification of developing complications in the majority of cases [5], COHb may improve earlier and bedside prediction of a failing oxygenator. In our study, COHb was predictive for oxygenator change irrespective of the underlying cause of oxygenator dysfunction which strengths the consideration of COHb as a global marker of oxygenator performance. Third, plasma free hemoglobin is a valuable marker for hemolysis, but was not available for this analysis, as it is not routinely measured at our institution. Haptoglobin was also only measured in a few cases so that the lack of data would have been so substantial that the analysis does not gain valuable information.

Finally, the retrospective single-center design may hinder generalizing the results, as the decision and time point of oxygenator change is based on local experience. Specifying and protocolizing clear indications for oxygenator change and evaluation of COHb performance in this setting to define clear cut-off values are priorities for further, preferably prospective research efforts.

## Conclusion

In ECMO patients, rising COHb levels are predictive of reduced oxygenator performance and imminent oxygenator change. This data advocate for integrating this readily available POC marker of hemolysis into routine monitoring to raise awareness and to earlier anticipate potential oxygenator failures to avoid emergency circuit changes. Further prospective trials are needed to identify reasonable cut-offs that can then be added to clinical algorithms to consider a change of oxygenator.

### Supplementary Information

**Additional file 1: Figure S1.** Conceptual Model. **Figure S2.** Histogram of COHb

## Data Availability

Ethics regulations do not allow data sharing from this article.

## Abbreviations

CI: Confidence interval
CO: Carbon monoxide
COHb: Carboxyhemoglobin
ECMO: Extracorporeal membrane oxygenation
*F*COHb: Fraction of total hemoglobin (ctHb) that is present as carboxyhemoglobin (COHb)
fHb: Free plasma hemoglobin
F_s_O_2_: Fraction of delivered oxygen by the sweep gas
INR: International normalized ratio
IQR: Interquartile range
SOFA: Sequential Organ Failure Assessment
LDH: Lactate dehydrogenase
NO: Nitric oxide
iNO: Inhalative nitric oxide
PTT: Partial thromboplastin time
pO_2_: Partial pressure of oxygen
PpostO_2_: Partial pressure of oxygen in post-oxygenator blood
TT: Thrombin time
V-V: Veno-venous
V-A: Veno-arterial
HO-1: Heme-oxygenase-1
POC: Point of care
