# Supplementary material for: Carboxyhemoglobin predicts oxygenator performance and imminent oxygenator change in extracorporeal membrane oxygenation
Source: Intensive Care Med Exp. 2024 Apr 24;12:41. doi: 10.1186/s40635-024-00626-7 (PMC11043307; doi:10.1186/s40635-024-00626-7)
Supplement: Supplementary file 1 — Additional file 1: Figure S1. Conceptual Model. Figure S2. Histogram of COHb [file 40635_2024_626_MOESM1_ESM.pdf]

Supplementary Material

Carboxyhemoglobin Predicts Oxygenator Performance and Imminent Oxygenator Change in Extracorporeal Membrane Oxygenation

Rolf Erlebach, Alix Buhlmann, Rea Andermatt, Benjamin Seeliger, Klaus Stahl, Christian Bode, Reto Schuepbach, Pedro David Wendel-Garcia, Sascha David, the BonHanZA (Bonn-Hannover-Zurich-ARDS) study group

Table of contents

Figure S1: Conceptual Model 3

Figure S2: Histogram of COHb 4

Figure S1: Conceptual Model (Directed Acyclic Graph)

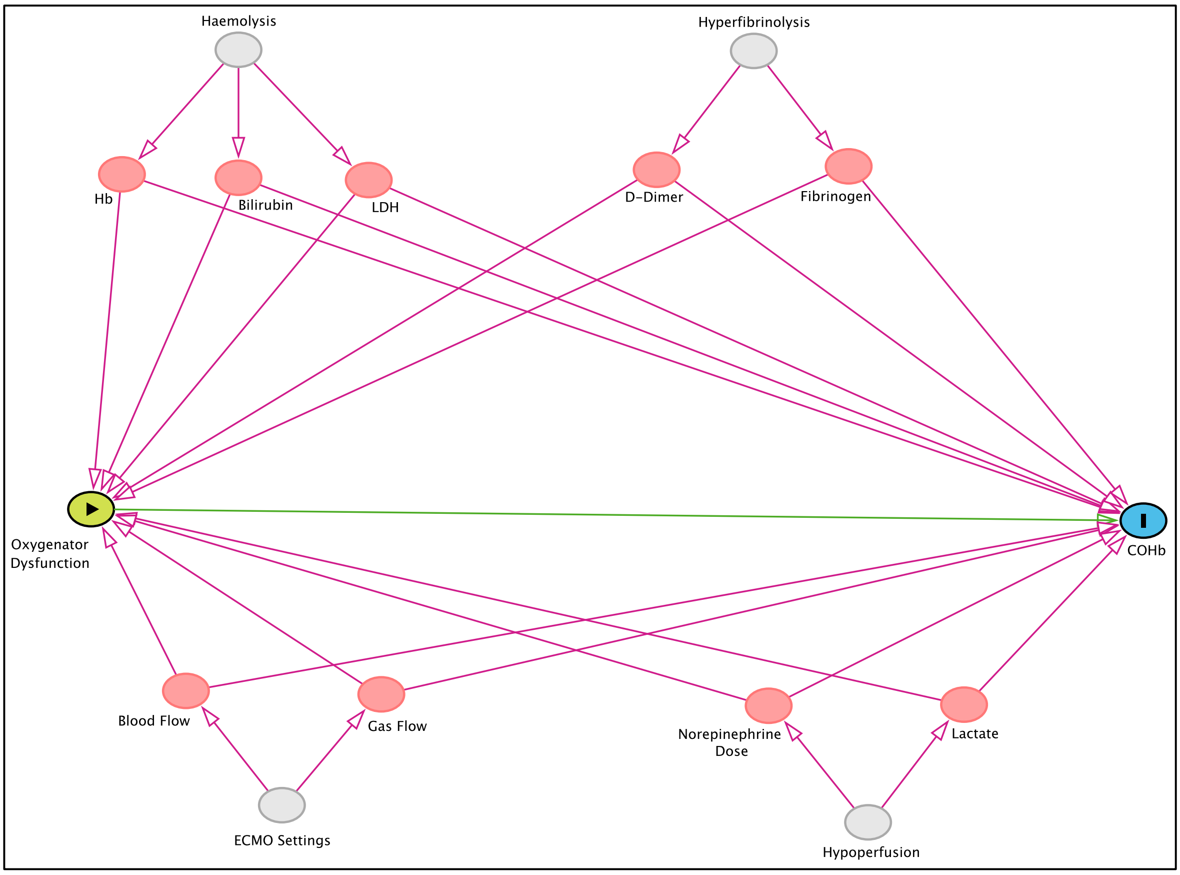

Directed acyclic graph depicting the conceptual model of causal association between oxygenator dysfunction and COHb. Oxygenator dysfunction (exposure) and its directed ancestors are coloured in green, whereas COHb (outcome) and its direct ancestors are coloured in blue. Ancestors of both oxygenator dysfunction and COHb are coloured in pink. Arrows present a directed causal and hierarchical relationship between two variables.

Abbreviations: COHb – carboxyhemoglobin, ECMO – extracorporeal membrane oxygenation, Hb – hemoglobin, LDH – lactate dehydrogenase.

Figure S2: Histogram of COHb

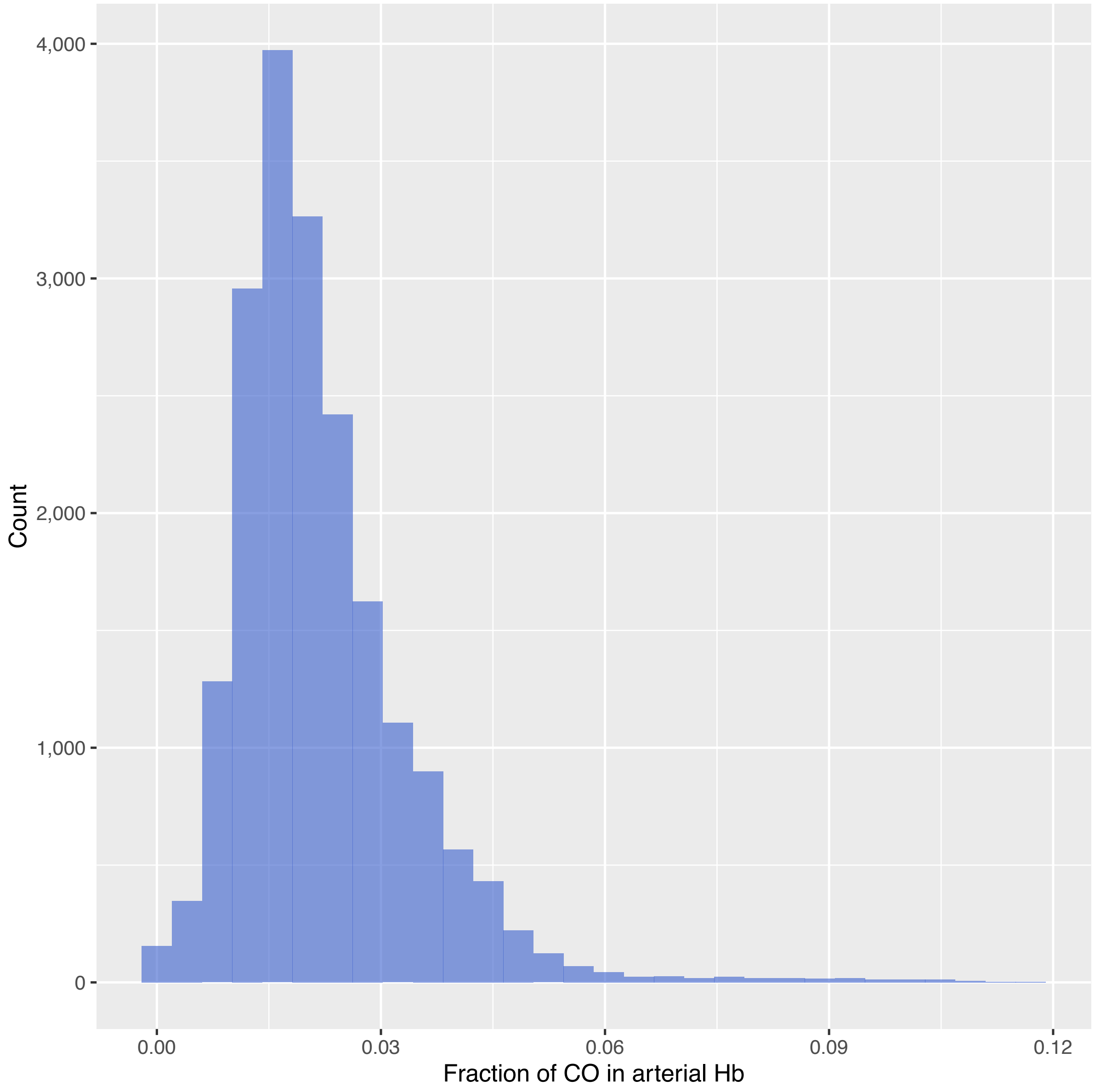

Histogram of arterial fraction of carboxyhemoglobin
